# Endocannabinoid basis of personality—Insights from animal model of social behavior

**DOI:** 10.3389/fphar.2023.1234332

**Published:** 2023-08-16

**Authors:** Natalya M. Kogan, Dilorom Begmatova, Liudmila Vinnikova, Sergey Malitsky, Maxim Itkin, Eyal Sharon, Artem Klinov, Jonathan Gorelick, Igor Koman, Zvi Vogel, Raphael Mechoulam, Albert Pinhasov

**Affiliations:** ^1^ Department of Molecular Biology, Ariel University, Ariel, Israel; ^2^ The Institute of Personalized and Translational Medicine, Ariel University, Ariel, Israel; ^3^ Institute of Drug Research, Hebrew University, Jerusalem, Israel; ^4^ Life Sciences Core Facilities, Weizmann Institute of Science, Rehovot, Israel; ^5^ Eastern Regional R&D Center, Kyriat Arba, Israel; ^6^ Department of Neurbiology, Weizmann Institute of Science, Rehovot, Israel; ^7^ Adelson School of Medicine, Ariel University, Ariel, Israel

**Keywords:** personality, endocannabinoidome, social behavior, dominance, submissiveness, endocannabinoid system, lipidomics, PUFA

## Abstract

**Rationale:** The endocannabinoid system is known to be involved in learning, memory, emotional processing and regulation of personality patterns. Here we assessed the endocannabinoid profile in the brains of mice with strong characteristics of social dominance and submissiveness.

**Methods:** A lipidomics approach was employed to assess the endocannabinoidome in the brains of Dominant (Dom) and Submissive (Sub) mice. The endocannabinoid showing the greatest difference in concentration in the brain between the groups, docosatetraenoyl ethanolamine (DEA), was synthesized, and its effects on the physiological and behavioral responses of Dom and Sub mice were evaluated. mRNA expression of the endocannabinoid receptors and enzymes involved in PUFA biosynthesis was assessed using qRT-PCR.

**Results:** Targeted LC/MS analysis revealed that long-chain polyunsaturated ethanolamides including arachidonoyl ethanolamide (AEA), DEA, docosatrienoyl ethanolamide (DTEA), eicosatrienoyl ethanolamide (ETEA), eicosapentaenoyl ethanolamide (EPEA) and docosahexaenoyl ethanolamide (DHEA) were higher in the Sub compared with the Dom mice. Untargeted LC/MS analysis showed that the parent fatty acids, docosatetraenoic (DA) and eicosapentaenoic (EPA), were higher in Sub vs. Dom. Gene expression analysis revealed increased mRNA expression of genes encoding the desaturase FADS2 and the elongase ELOVL5 in Sub mice compared with Dom mice. Acute DEA administration at the dose of 15 mg/kg produced antinociceptive and locomotion-inducing effects in Sub mice, but not in Dom mice. Subchronic treatment with DEA at the dose of 5 mg/kg augmented dominant behavior in wild-type ICR and Dom mice but not in Sub mice.

**Conclusion:** This study suggests that the endocannabinoid system may play a role in the regulation of dominance and submissiveness, functional elements of social behavior and personality. While currently we have only scratched the surface, understanding the role of the endocannabinoid system in personality may help in revealing the mechanisms underlying the etiopathology of psychiatric disorders.

## 1 Introduction

Since the discovery of Δ^9^-tetrahydrocannabinol (THC) over 60 years ago, the main psychoactive component in cannabis, and subsequent identification of cannabinoid receptors and their endogenous agonists, the functional role of the endocannabinoid system (ECS) in animals has gained greater recognition (Rezende et al., 2023). Many fatty acid amides and related compounds, structurally similar to the main endocannabinoid, arachidonoyl ethanolamide (AEA), have been identified in the brain, yet with only a handful actually studied (Alharthi et al., 2018). Those that were studied showed diverse effects, including anti-inflammatory and neuroprotective activities (Donvito et al., 2018). Cannabinoid receptors and their endogenous agonists are found throughout the body and are involved in a range of physiological processes including inflammation (Eljaschewitsch et al., 2006), pain (Bruni et al., 2018), immune response (Rahaman and Ganguly, 2021), regulation of sleep (Kesner and Lovinger, 2020) and appetite (Aguilera Vasquez and Nielsen, 2022). The ECS is also important for learning and memory (Morena and Campolongo, 2014), as well as regulation of emotional, motivational, and cognitive functions (Viveros et al., 2007; Magen et al., 2009; Schechter et al., 2013; Morena and Campolongo, 2014; Manduca et al., 2015; Murlanova et al., 2022a). Modulation of the ECS has also been shown to be protective against neurodegenerative disorders (Kogan and Mechoulam, 2007). Further, altered levels of endocannabinnoids have been detected in neuropsychiatric disorders (Schaefer et al., 2014; Romero-Sanchiz et al., 2016; Wingenfeld et al., 2018). Some elements of the ECS have been proposed for use as biomarkers and even as potential targets for the treatment of anxiety and depression (Chadwick et al., 2020; Gallego-Landin et al., 2021; Rana et al., 2021; Bright and Akirav, 2022). Accumulating evidence also suggests a role for the ECS in borderline personality disorder, antisocial behavior, post-traumatic stress disorder, and manic-depressive disorder (Kolla and Mishra, 2018; Wingenfeld et al., 2018; Ferber et al., 2020; Ho and Kolla, 2022; Topuz et al., 2022; Spohrs et al., 2023).

The ECS is involved also in normal emotional processes (Viveros et al., 2007; Morena and Campolongo, 2014; Arndt and de Wit, 2017). AEA and 2-arachidonoylglycerol (2-AG) have been shown to play a role in regulation of social behavior (Wei et al., 2017). Anandamide levels are elevated in rat striatum after meeting an unfamiliar animal, compared with normal levels when a familiar animal was met (Marco et al., 2011). Knockout mutation of fatty acid amide hydrolase (FAAH), responsible for anandamide degradation, enchanced social interactions in mice (Cassano et al., 2011). Anandamide was found to reduce social anxiety (File and Seth, 2003). A “rough-and-tumble” social play in juvenile rats was found to be associated with increased levels of anandamide (Trezza et al., 2012) and 2-AG (Manduca et al., 2015). Mouse knockout mutation of the hydrolytic enzyme monoacylglycerol lipase produced impaired conditioned place preference to social stimuli; and prolonged social contact for 6 h was found to stimulate 2-AG mobilization (Wei et al., 2016). However, the role of other members of the ECS in social behavior remains unclear.

In this work, endocannabinoid brain profiles and responses to a selected endocannabinoid were evaluated in mouse models of social behavior developed through multiple generations of selective breeding based on social interaction food competition dominant-submissive relationship (DSR) paradigm (Feder et al., 2010). These animals exhibit strong and stable characteristics of dominance (Dom strain) or submissiveness (Sub strain), possess inherited stress resilience or vulnerability, respectively (Murlanova et al., 2022b), and differentially respond to psychotropic agents (Nesher et al., 2013).

## 2 Materials and methods

### 2.1 Animals

Three-month-old dominant (Dom) and submissive (Sub) male mice selectively bred over 47 generations (Feder et al., 2010; Nesher et al., 2013) and commercially available wild-type ICR (CD-1) mice (Inotiv, Israel) were used in this study. Animals were given standard laboratory chow (Teklad, Inotiv, Israel) and water *ad libitum* in a colony room maintained on a 12:12 L:D cycle (lights on 07:00-19:00 h). The present study received approval by the Israel Ministry of Health and the Ariel University Institutional Animal Care and Use Committee (permission # AU-IL-2305-107).

### 2.2 Endocannabinoid levels analyses

#### 2.2.1 Targeted whole brain lipidome analysis

Extraction and analysis of endocannabinoids were performed as described elsewhere (Malitsky et al., 2016; Gnainsky et al., 2022) with the following modifications: mouse brain (*n* = 7 per group of Sub female, Dom female, Sub male, and Dom male) was lyophilized overnight at −110°C, homogenized, and transferred to separate 2 mL Eppendorf tubes (10 mg homogenate per tube). Metabolites were extracted with solution consisting of 1 mL of a pre-cooled (−20°C) methanol:methyl-tert-butyl-ether (MTBE, 1:3, v/v) solution containing the following internal standards: 0.1 μg/mL of phosphatidylcholine (17:0/17:0) (Avanti Polar Lipids, Alabaster, AL, United States), 0.4 μg/mL of phosphatidylethanolamine (17:0/17:0) (Avanti Polar Lipids, Alabaster, AL, United States), and 2 μg/mL (arachidonyl-1′-hydroxy-2′-propylamide, a stable synthetic AEA derivative synthesized by us). The tubes were vortexed and then sonicated for 30 min in an ice-cold sonication bath (with brief vortexing every 10 min). UPLC-grade water:methanol (3:1, v/v) solution (0.5 mL) was then added to tubes followed by centrifugation at 17,950 x g. The upper organic phase was transferred to a fresh tube and the polar phase was re-extracted as described above with 0.5 mL of MTBE. Both organic phases were combined, dried in SpeedVac (Savant, Thermo Scientific, United States), and stored at −80°C. For analysis, dried lipid extracts were re-suspended in 100 μL mobile phase B (see below) and centrifuged again at 17,950 × g at 4°C for 10 min and transferred to HPLC injection vials.

Chromatographic separation was performed on an ACQUITY UPLC BEH C8 column (2.1 × 100 mm, i.d., 130 Å, 1.7 μm; Waters, Israel). Mobile phase A: H_2_O:acetonitrile:2-propanol 46:38:16 (v/v/v), 1% 1.0 M ammonium acetate, 0.1% acetic acid. Mobile phase B: H_2_O:acetonitrile:2-propanol 1:69:30 (v/v/v), 1% 1.0 M ammonium acetate, 0.1% acetic acid.

The column was maintained at 40°C with a mobile phase flow rate of 0.4 mL/min. Mobile phase A was run for 1 min at 100%, then gradually reduced to 25% at 12 min, following a decrease to 0% at 16 min. Then, mobile phase B was run at 100% until 21 min and mobile phase A was set to 100% at 21.5 min. Finally, the column was equilibrated at 100% A until 25 min. Endocannabinoids were measured by UPLC-ESI-MS/MS equipped with the ACQUITY UPLC I-Class PLUS system (Waters, Israel). The MS detector (Xevo TQ-XS; Waters, Israel) was equipped with an ESI source and measurement was performed in positive ionization mode using MRM. MS parameters were as follows: the source and de-solvation temperatures were maintained at 150°C and 400°C, respectively. The capillary voltage was set to 1.5 kV. Nitrogen was used as the de-solvation gas and cone gas at flow rates of 800 L/h and 150 L/h, respectively.

#### 2.2.2 Untargeted whole-brain lipidome analysis

After the sample preparation (as described above, the same brain extracts have been used for targeted and for the untargeted analyses), untargeted lipidomics was performed by UPLC-ESI-MS/MS equipped with the ACQUITY UPLC I-Class PLUS system (Waters, Milford, MA, United States). The LC conditions were as described in the previous section. The mass analyzer (Vion IMS QTof; Waters, Israel) was equipped with an ESI source and parameters were as follows: the source and de-solvation temperatures were maintained at 120°C and 450°C, respectively. The capillary voltage was set to 3.0 kV and 2.0 kV for positive and negative ionization mode, respectively; cone voltage was set for 40 V. Nitrogen was used as the de-solvation and cone gas at a flow rate of 800 L/h and 30 L/h, respectively. The mass spectrometer was operated in full scan HDMS^E^ resolution mode over a mass range of 50–2000 Da. For the high-energy scan function, a collision energy ramp of 20–80 eV was applied and for the low energy scan function, −4 eV was applied. Data processing was performed with Progenesis QI software (Nonlinear Dynamics, Newcastle upon Tyne, United Kingdom). The lipids were identified by comparing the masses and the fragments to databases: HMDB (Human Metabolome Database) (Wishart et al., 2022), ChemSpider (Pence and Williams, 2010), and LipidBlast (Kind et al., 2013).

### 2.3 Quantitative RT-PCR analysis

Total RNA was isolated from whole brains (*n* = 5 per group) of male Sub and Dom mice utilizing an EZ-RNA Total RNA Isolation Kit according to the manufacturer’s guidelines (Biological Industriess, Israel). RNA was eluted in a volume of 100 μL and RNA concentration was determined by NanoDrop One Microvolume UV-Vis Spectrophotometer (Thermo Scientific, Waltham, MA, United States). cDNA synthesis was performed on 2000 ng (two reactions per sample) of total RNA employing a Verso cDNA Synthesis Kit (Thermo Scientific, Waltham, MA, United States) according to the manufacturer guidelines. Primers were designed by Integrated DNA Technologies (Coralville, IA, United States) employing FAM/ZEN/IBFQ configuration:

**Table:** 

Gene query	Assay ID	Ref seq
*Gapdh*	Mm.PT.39a.1	NM_008084 (1)
*Cnr1*	Mm.PT.58.30057922	NM_007726 (1)
*Cnr2*	Mm.PT.58.41156189	NM_009924 (1)
*Gpr18*	Mm.PT.58.41931372.g	NM_182806 (1)
*Gpr55*	Mm.PT.58.41232402	NM_001033290 (1)
*Trpv1*	Mm.PT.58.13426135	NM_001001445 (1)
*Fads1*	Mm.PT.58.30329182	NM_146094 (1)
*Fads2*	Mm.PT.58.41319081	NM_019699 (1)
*Fads3*	Mm.PT.58.6548462	NM_021890 (1)
*Elovl1*	Mm.PT.58.6057973	NM_001039175 (2)
*Elovl2*	Mm.PT.58.14130708	NM_019423 (1)
*Elovl3*	Mm.PT.58.5393956	NM_007703 (1)
*Elovl4*	Mm.PT.58.5889776	NM_148941 (1)
*Elovl5*	Mm.PT.58.43010575	NM_134255 (1)
*Elovl6*	Mm.PT.58.29389101	NM_130450 (1)

The *Gapdh* gene was used as an endogenous control. RT-PCR was performed on an Azure Cielo Real-Time PCR system (Azure Biosystems, Dublin, CA, United States) using Prime-Time qPCR Primer Assays (Integrated DNA Technologies, Coralville, IA, United States) in a 20 μL reaction mix containing 3 μL of cDNA, 10 μL of 2× master mix buffer, 1 μL of Prime-Time qPCR Probe assay mix (containing primers and probe), and 6 μL of water. The amplification program was as follows: 95°C for 3 min, 44 cycles of 95°C for 10 s, and 60°C for 30 s.

### 2.4 DEA synthesis

**FIGURE 1:**
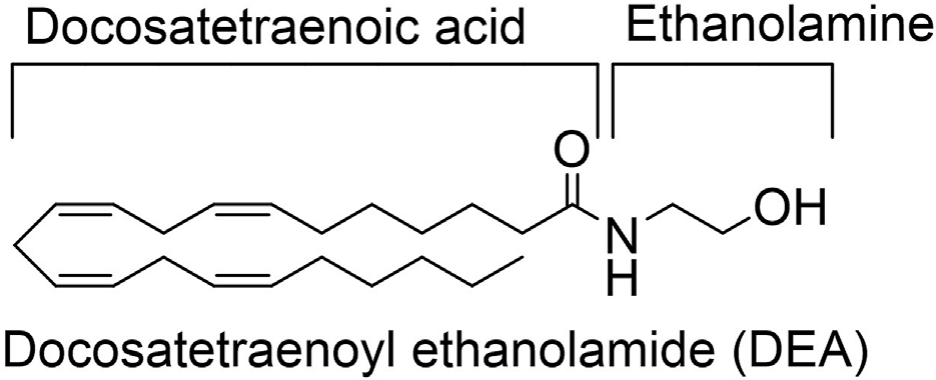
DEA structure.

DEA was synthesized and purified as previously described. The structure is depicted at Figure 1 (Hanus et al., 1993).

### 2.5 Assessment of analgesic and behavioral effects of DEA in mouse models of dominance and submissiveness

DEA was administered (5, 10, or 15 mg/kg i.p., *n* = 5) to male Sub (*n* = 5) and Dom (*n* = 5) behavior assessment-naïve mice. These concentrations have been chosen, as doses higher than 15 mg/kg produce psychotropic response, as has been previously observed (Hanus et al., 1993; Barg et al., 1995). One hour after injection, the animals were subjected to Elevated Plus Maze (EPM) and Hot Plate (HP) tests.

#### 2.5.1 Hot plate (HP) test

The HP test was employed to assess nociception. The hot plate was pre-heated to 55°C. After placement on the hot plate, mouse was removed immediately with appearance of a nociceptive response such as hind paw licking, hind paw flicking, vocalization, or jumping. Mice showing no such response within 15 s were removed from the apparatus to prevent tissue damage.

#### 2.5.2 Elevated Plus Maze (EPM)

Anxiety-like behavior was assessed in the EPM test using EthoVision-XT video tracking software (Noldus, Wageningen, Netherlands) as described previously (Nesher et al., 2013). Briefly, each mouse was placed in the center of EPM and was allowed to explore the apparatus for 5 min. Locomotor (distance travelled and velocity) and exploratory (number of entries into open and closed arms, as well as dwell time spent in open and closed arms) activities were then software tallied.

#### 2.5.3 Dominant-submissive relationship test (DSR)

The DSR test is a repeated food competition paradigm developed to study submissiveness as a model of depressive-like behavior and dominance as a model of manic-like behavior (Malatynska and Knapp, 2005; Malatynska et al., 2007; Pinhasov et al., 2023). Dominant (Dom strain) and submissive (Sub strain) mice resulting from DSR assessment were selectively bred over 47 generations based on their performances in the DSR test (Feder et al., 2010; Nesher et al., 2015; Basil et al., 2018; Gross et al., 2018). DSR tests were conducted over nine consecutive days. Mice were fasted for a 16-h period before testing with water provided *ad libitum*. During a 5-min DSR session, milk drinking times for each mouse were manually recorded. DEA (5 mg/kg i.p.) or vehicle (1:1:18 ethanol:Tween 80:saline) i.p. was injected 30 min before placing the pair of mice into the DSR arena.

### 2.6 Statistical analysis

All the data represent means ± SEM. The following annotations apply to all presented data: *p < 0.05, **p < 0.01, ***p < 0.001, ****p < 0.0001 vs. control. For metabolomic tests (*n* = 7 mice per group), a Mann-Whitney test was performed to assess the difference in every metabolite by relative abundance measurement in Dom groups to the values in the corresponding Sub groups (Dom males vs. Sub males, Dom females vs. Sub females). For HP test and EPM tests (*n* = 5 per group), the groups were compared by ordinary one-way ANOVA followed by Dunnett’s multiple comparisons test, treatment groups were compared to the vehicle group. For DSR test (*n* = 5 per group) the groups were compared by repeated measurements two-way ANOVA followed by Šídák’s multiple comparisons test.

## 3 Results

### 3.1 Endocannabinoid brain levels differ between Sub and Dom mice

#### 3.1.1 Targeted whole-brain lipidome analysis revealed elevated levels of long-chain polyunsaturated ethanolamines in Sub mice

Triple-quadrupole (tripleQ) targeted LC-MS lipidomic analysis of the brain samples revealed several differences between the Dom and Sub populations. The levels of long-chain ethanolamides, specifically C20 (eicosa-) and C22 (docosa-), especially with 3–5 double bonds, were found to be elevated in Sub compared with Dom (Table 1). Specifically, AEA (20:4), DTEA (22:3), EPEA (20:5), and DHEA (22:6) were significantly higher in male Sub compared with male Dom, while ETEA (20:3) was higher in female Sub compared with female Dom. DEA (22:4) levels were elevated in both male and female Sub compared with respective Dom groups. No significant difference among Dom and Sub mice in the content of monounsaturated DEEA (22:1), saturated DSEA (22:0), or medium-chained fatty acid ethanolamides was observed (Table 1). In addition, no significant difference was observed between the groups in either males or females regarding the levels of other endocannabinoids: glycerols of the tested fatty acids (2-arachidonoyl glycerol (2-AG), 2-palmitoyl glycerol (2-PG), 2-linoleoyl glycerol (2-LG)) and fatty-acyl-amino acids (arachidonoyl glycine (ARA-G), arachidonoyl serine (ARA-S), oleoyl taurine (OT), oleoyl alanine (OA) and oleoyl glycine (OG)) (Table 1).

**TABLE 1:** Endocannabinoid levels in whole mouse brain. The endocannabinoid levels were measured by the TripleQ LCMS in the whole-brain extracts of Sub and Dom male and female mice. The data is presented as relative abundance units. *p < 0.05, **p < 0.01 by Mann-Whitney test for Dom females vs. Sub females and for Dom males vs. Sub males.

Compound (#carbons: #Double bonds)	Sub females	Dom females	Sub males	Dom males
Lau_EA (12:0)	130,270 ± 50,032	179,179 ± 32,540	85,582 ± 42,742	38,957 ± 16,226
MEA (14:0)	87,706 ± 21,004	109,745 ± 12,861	73,100 ± 17,759	43,388 ± 11,344
PDEA (15:0)	9,385 ± 1,029	10,482 ± 1,112	8,930 ± 1,230	7,726 ± 1,011
PEA (16:0)	3,904,268 ± 250,633	4,381,209 ± 295,502	3,388,942 ± 121,783	2,927,222 ± 383,324
SEA (18:0)	1,777,579 ± 209,355	1,985,862 ± 112,493	1,498,847 ± 85,670	1,248,505 ± 215,990
Oleamide (18:1)	682,274 ± 54,220	777,163 ± 65,404	664,335 ± 52,469	514,705 ± 67,482
OEA (18:1)	523,425 ± 40,149	572,977 ± 53,261	491,333 ± 29,673	377,573 ± 56,308
VEA (18:1)	1,009,128 ± 66,541	1,028,019 ± 65,998	944,019 ± 26,827	721,695 ± 94,350
LEA (18:2)	51,525 ± 4,968	65,264 ± 4,409	73,580 ± 14,669	53,341 ± 9,667
**ETEA (20:3)**	**400 ± 64**	248 ± 43*	366 ± 50	332 ± 36
**AEA (20:4)**	66,311 ± 4,968	61,753 ± 1,749	**71,014 ± 10,074**	42,265 ± 5,342*
**EPEA (20:5)**	468 ± 71	347 ± 68	**497 ± 50**	274 ± 57*
DSEA (22:0)	455,198 ± 43,173	524,555 ± 47,381	360,490 ± 20,031	420,025 ± 66,381
DEEA (22:1)	225,480 ± 16,356	212,501 ± 18,153	174,229 ± 12,973	139,264 ± 22,873
**DTEA (22:3)**	411 ± 64	247 ± 43	**341 ± 70**	114 ± 30*
**DEA (22:4)**	**12,982 ± 1,220**	9,215 ± 782**	**10,714 ± 1,498**	6,246 ± 926*
**DHEA (22:6)**	40,724 ± 2,018	43,505 ± 2,029	**41,541 ± 3,452**	25,571 ± 5,212*
ARA-G	7,744 ± 872	9,755 ± 1,212	9,103 ± 1,461	6,948 ± 1,057
ARA-S	2,744 ± 368	3,212 ± 463	2,976 ± 456	2,303 ± 259
OT	33,085 ± 4,299	35,237 ± 4,525	34,897 ± 4,512	27,019 ± 4,923
OG	12,764 ± 2,167	20,332 ± 3,939	14,175 ± 2,019	11,858 ± 2,397
OS	62,441 ± 13,307	94,953 ± 22,852	70,820 ± 12,249	58,796 ± 9,994
OA	6,486 ± 601	8,829 ± 1,005	6,512 ± 749	6,828 ± 1,039
2-PG	119,994 ± 9,326	146,383 ± 8,885	134,628 ± 8,630	125,548 ± 11,015
2-LG	76,150 ± 5,696	99,045 ± 15,271	97,601 ± 10,895	110,010 ± 12,065
2-AG	5,220,018 ± 436,410	6,215,627 ± 504,893	5,362,587 ± 111,369	5,168,100 ± 662,765

The significant changes are bold.

#### 3.1.2 Untargeted whole-brain lipidome analysis revealed higher levels of parental fatty acids for DEA and EPEA in Sub mice

Untargeted analysis using high-resolution (HR) LC-MS identified 318 metabolites. When the cutoff criteria for important metabolites was set for *p* < 0.05 and fold change >1.25 in both male and female groups, the Progenesis QI software identified four compounds. Phosphatidylcholines (PC 40:2 and PC 40:3), DA (the precursor of DEA), and EPA (the precursor of EPEA) were significantly higher in both male and female Sub compared with Dom mice (Table 2). The ethanolamides observed in tripleQ were not observed in the untargeted analysis, as their levels in the brain were below the detection limit for HRLC-MS.

**TABLE 2:** The most different endogenous lipids in the whole brain extracts of Sub and Dom male and female mice, as measured by the High-Resolution LCMS. The data is presented as relative abundance units. *p < 0.05, **p < 0.01 by Mann-Whitney test for Dom females vs. Sub females and for Dom males vs. Sub males.

Compound (#carbons: #Double bonds)	Sub females	Dom females	Sub males	Dom males
DA (22:4)	55.22 ± 1.36	43.11 ± 2.66**	55.51 ± 3.98	37.39 ± 4.43*
EPA (20:5)	2.61 ± 0.15	2.09 ± 0.15*	2.94 ± 0.19	2.20 ± 0.27*
PC (40:3)	60.61 ± 5.56	38.82 ± 2.53**	53.88 ± 5.01	34.68 ± 4.16*
PC (40:2)	1,147.50 ± 96.53	809.51 ± 67.43*	1,018.18 ± 61.84	704.69 ± 91.89*

### 3.2 mRNA levels of key enzymes of long-chained PUFA biosynthesis were higher in sub mice

**FIGURE 2:**
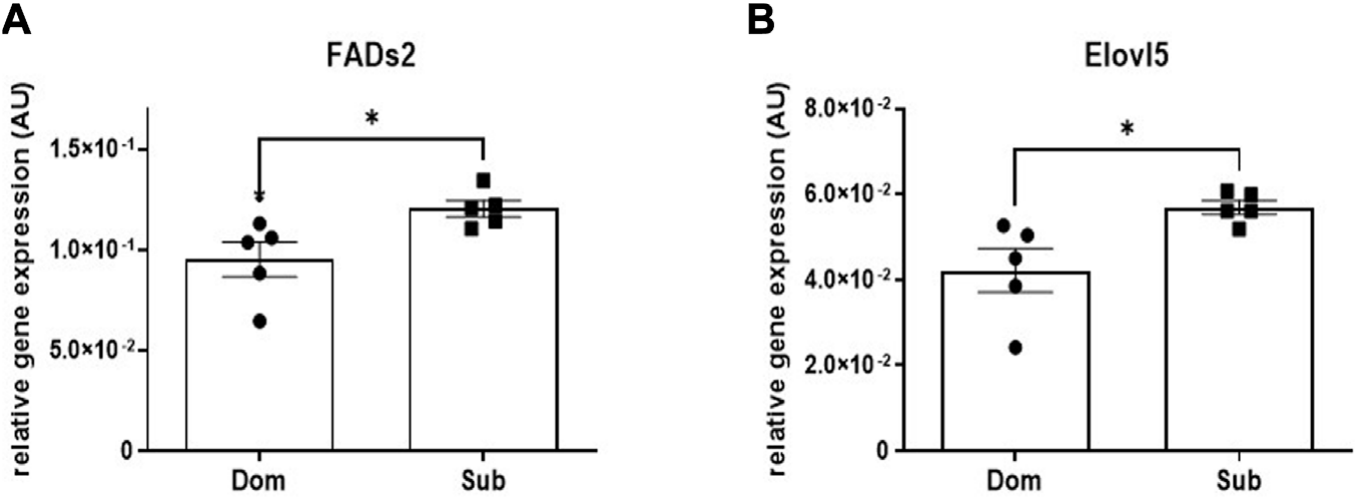
Whole brain levels of gene transcription in Sub and Dom mice. **(A)** *Fads2*; **(B)** *Elovl5*. mRNA expression was measured by the real-time RT-PCR in whole-brain extracts of Sub and Dom male mice. The data is presented as relative abundance. *p < 0.05 by Mann-Whitney test.

A significant increase in the mRNA expression of Fatty Acid Desaturase 2 (*Fads2*, 30%), as well as in the Elongation of Very Long-Chain Fatty Acids Protein 5 (*Elovl5*, 22%) was observed in brains of Sub mice compared with Dom mice (Figure 2). No significant difference in mRNA expression levels were observed among known cannabinoid receptors including *Cnr1*, *Cnr2*, *Gpr55*, *Gpr18*, and *Trpv1* (Supplementary Figure S2).

### 3.3 Effects of DEA treatment on animal behavior

Based on the endocannabinoid profiles observed, DEA, which was found as the most different between Dom and Sub male and female mice, was evaluated on animal responses in behavioral tests aimed to assess nociception (HP), anxiety (EPM) and social behavior (DSR).

#### 3.3.1 Acute DEA administration exhibited dose- and phenotype- dependent anti-nociceptive effects

**FIGURE 3:**
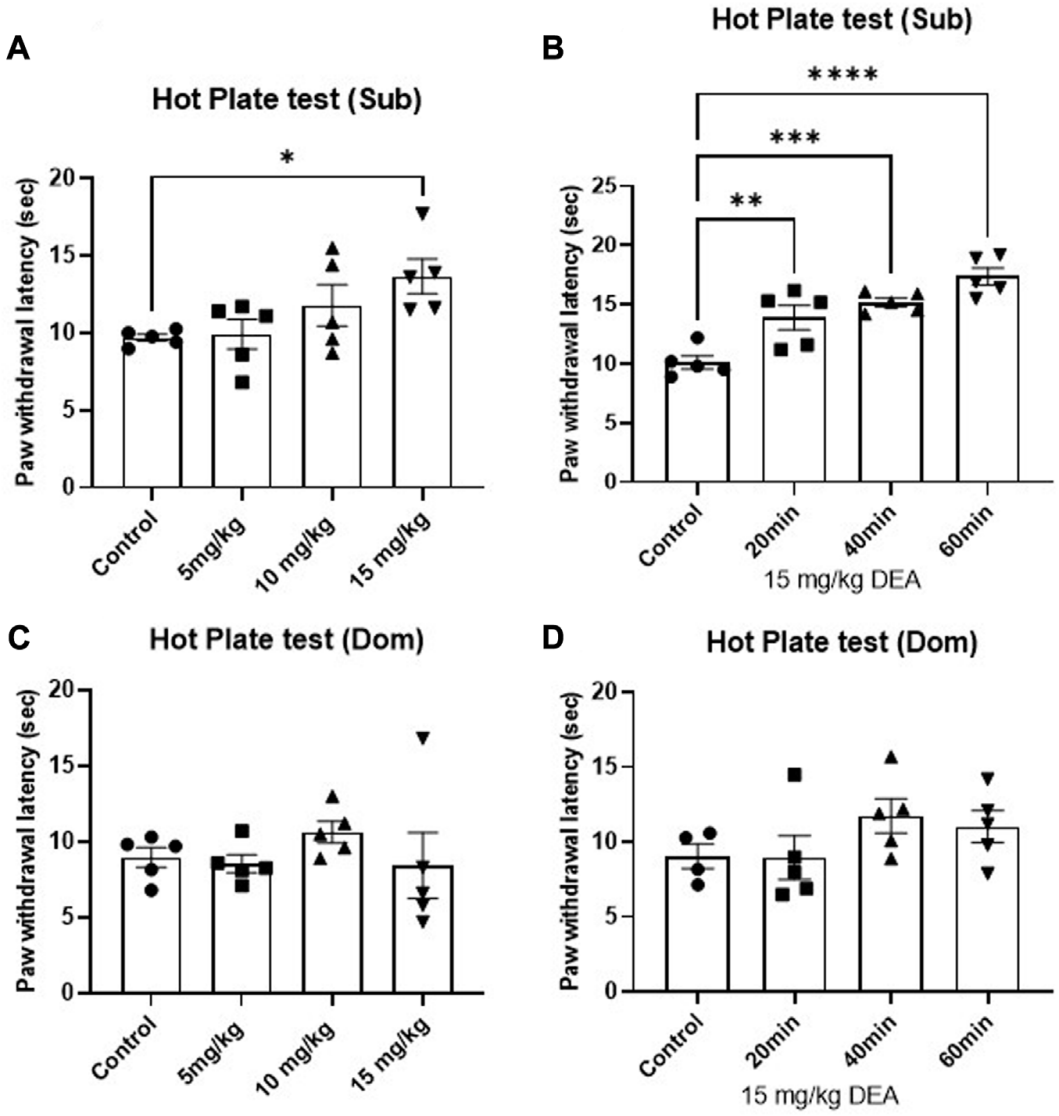
Effects of acute DEA treatment on thermal hyperalgesia induced by a hot plate in Sub and Dom mice. **(A)** Effect of DEA (5, 10, and 15 mg/kg i.p.) in Sub mice measured 40 min after the injection. **(B)** Effect of DEA (15 mg/kg i.p.) on Sub mice measured 20, 40 and 60 min after the injection. **(C)** Effect of DEA (5, 10, and 15 mg/kg i.p.) on Dom mice measured 40 min postinjection. **(D)** Effect of DEA (15 mg/kg i.p.) in Dom mice measured 20, 40 and 60 min after the injection. DEA was dissolved in 1:1:18 ethanol:Tween 80:saline. *n* = 5 per group. Control, vehicle only. All the data are represented as mean ± SEM. *p < 0.05, **p < 0.01, ***p < 0.001, ****p < 0.0001 vs. control, by regular one-way ANOVA followed by Dunnett’s multiple comparisons test.

The effect of DEA on nociception was tested using HP test. Single i.p. administration of DEA produced significant anti-nociceptive effects in Sub male mice. One-way ANOVA analysis demonstrates that among three tested doses (5, 10, 15 mg/kg), the highest tested dose showed a pronounced anti-nociceptive effect in male Sub mice (Figure 3A, *F* = 3.41, *p* = 0.043), which was sustained at 20, 40, and 60 min after drug administration (Figure 3B, *F* = 18.10, *p* < 0.0001). In contrast, no dose- or time-dependent effect of DEA at the tested doses was observed in Dom mice (Figure 3C, *F* = 0.69, *p* = 0.57 and Figure 3D, *F* = 1.42, *p =* 0.28).

#### 3.3.2 Acute DEA administration produced phenotype-dependent locomotion-inducing effects

**FIGURE 4:**
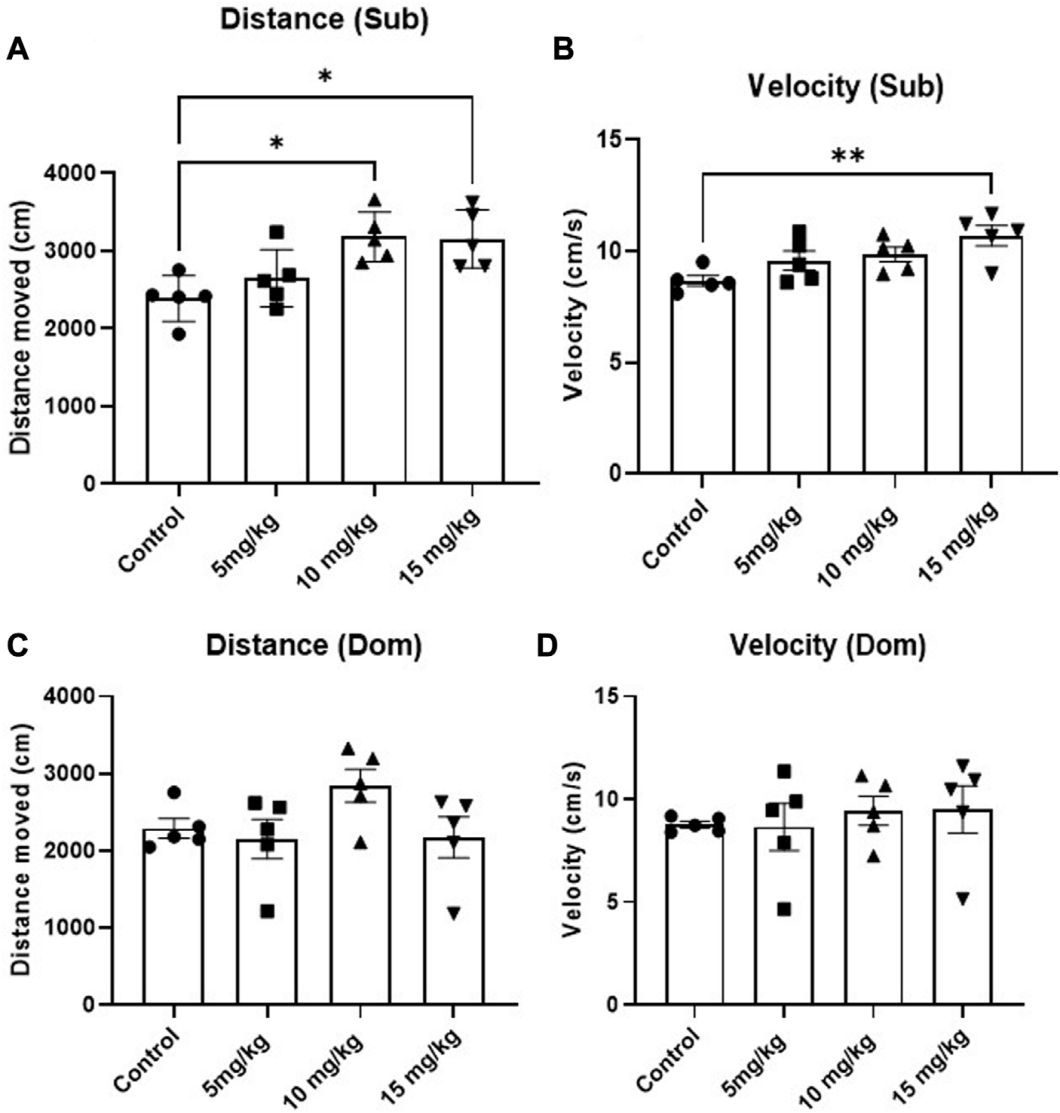
Effects of acute DEA (5, 10, and 15 mg/kg i.p.) treatment on locomotory activity in Elevated Plus Maze (EPM) in Sub and Dom mice. **(A)** Distance travelled in EPM, Sub mice. **(B)** Movement velocity in EPM, Sub mice. **(C)** Distance travelled in EPM, Dom mice. **(D)** Movement velocity in EPM, Dom mice. DEA was dissolved in 1:1:18 ethanol:Tween 80:saline. *n* = 5 per group. Control, vehicle only. All the data are represented as mean ± SEM. *p < 0.05, **p < 0.01, ***p < 0.001, ****p < 0.0001 vs. control, by regular one-way ANOVA followed by Dunnett’s multiple comparison test.

The anxiolytic-like and locomotion-inducing properties of DEA were evaluated using the EPM test. While no anxiolytic effect was observed, an acute, single i.p. administration of 15 mg/kg 1h before the test induced mouse locomotion in Sub mice compared with vehicle-treated animals (Figure 4A, *F* = 6.47, *p* = 0.005 and Figure 4B, *F* = 4.94, *p* = 0.013). In contrast, this phenomenon was not observed in Dom mice (Figure 4C, *F* = 2.16, *p* = 0.132 and Figure 4D, *F* = 0.25, *p* = 0.861). As for the preference between open and closed arms, while there appears to be an anxiolytic-like trend and elevated preference of open arms in both Sub and Dom mice, these differences are not statistically significant (Supplementary Figure S1).

#### 3.3.3 Subchronic DEA administration increased social dominance in a phenotype-dependent manner

**FIGURE 5:**
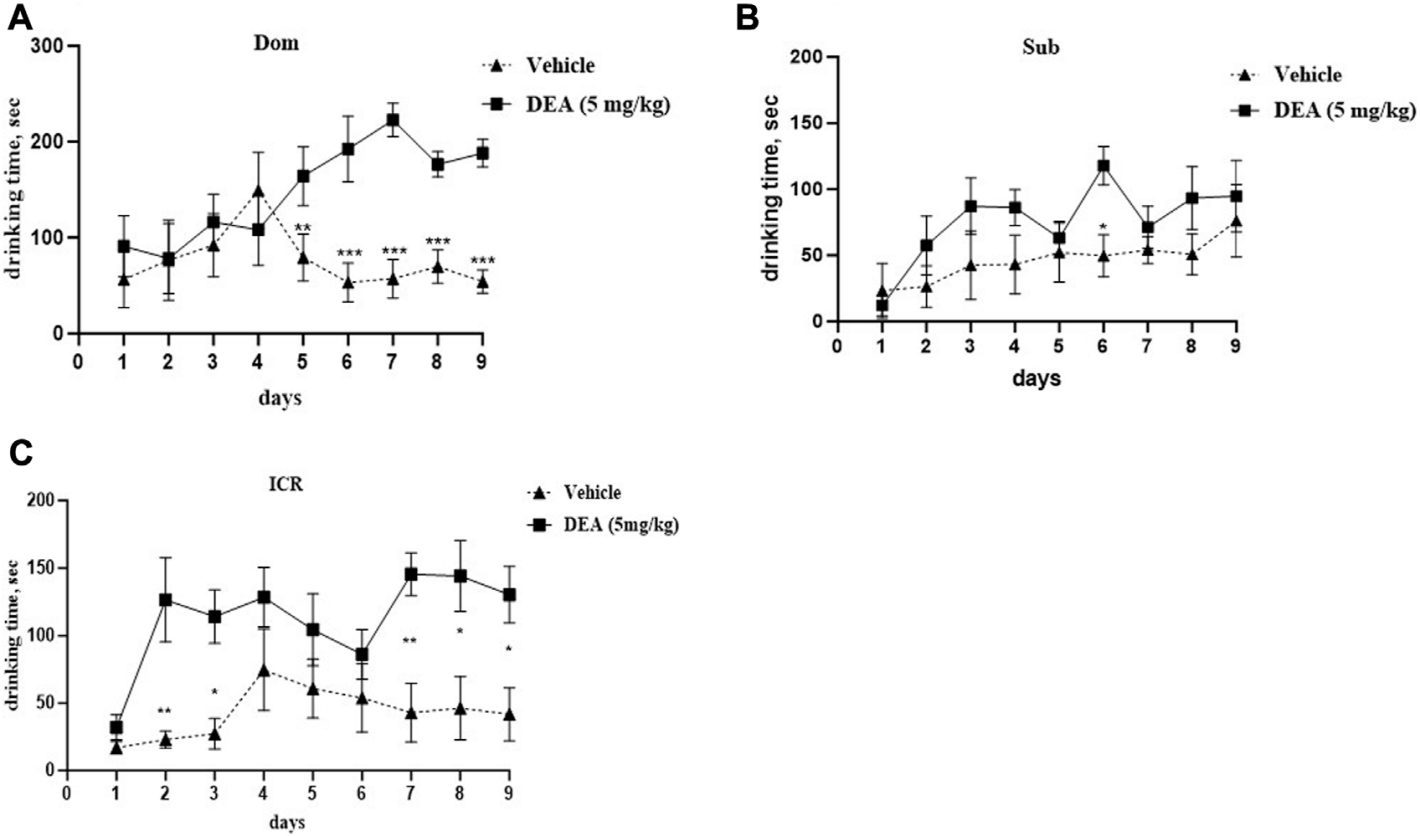
Effects of DEA (5 mg/kg, i.p.) treatment on animal behavior in the Dominant-Submissive Relationship (DSR) test. **(A)** Dom mice. **(B)** Sub mice, and **(C)** ICR mice. DEA was dissolved in 1:1:18 ethanol:Tween 80:saline. *n* = 5 per group. Controls, vehicle only. The duration of treatment lasted for 9 days. All the data are represented as mean ± SEM.*p < 0.05 vs. control, the groups were compared by repeated measurements two-way ANOVA followed by Šídák’s multiple comparisons test.

Two-way ANOVA analysis of the subchronic administration of DEA (5 mg/kg i.p., daily) demonstrated a significant increase in social dominance in Dom mice (Figure 5A, *F* (8,64) = 4.77, *p* = 0.0001). After treatment with DEA, Dom mice spent more time drinking over successive days, expulsing their vehicle-treated paired counterparts from the feeder, the difference which was statistically significant starting from day 5 of treatment and until the end of the experiment at day 9. This effect was not observed in Sub mice at the same tested dose (Figure 5B, *F* (8,64) = 1.004, *p* = 0.442). Interestingly, in ICR mice, DEA enhanced sociability, yet, to a lesser extent compared with the effect observed in Dom mice (Figure 5C, *F* (8,64) = 2.52, *p* = 0.019).

## 4 Discussion

In this work for the first time, we explored the endocannabinoidome in the brains of mice with strong and stable characteristics of social dominance and social submissiveness. Our evaluation revealed a number of interesting patterns: i) long-chain polyunsaturated ethanolamides, specifically C20 (eicosa-) and C22 (docosa-), were found to be higher in the brains of the Sub group compared with their Dom counterparts; ii) fatty acid precursors, DA and EPA, of the related ethanolamides, DEA and EPEA, were higher in Sub mouse brains compared to Dom; iii) the transcription levels of genes encoding key enzymes involved in polyunsaturated long-chained fatty acid biosynthesis, FADS2 and ELOVL5, were significantly higher in Sub mice brains; iv) acute DEA administration dose-dependently reduced nociception and induced locomotion in Sub mice only; v) subchronic DEA administration markedly induced dominance behaviors in a phenotype-dependent manner.

The endocannabinoidome encompasses structurally-related lipid mediators (Di Marzo and Wang, 2014; Piscitelli, 2015) involved in diverse biological mechanisms, with broad modulatory effects extending beyond the traditional endocannabinoid signaling pathways mediated by anandamide and 2-AG (Pacher, 2006; Pacher and Kunos, 2013; Pacher et al., 2020). Given that endocannabinoids play a crucial role in brain development, neural plasticity and function (Viveros et al., 2007), it is expected that changes in their concentrations would be associated with specific behavioral manifestations. Currently, the endogenous function of only AEA and 2-AG has been extensively investigated, along with their impacts on behavior (Wei et al., 2017; Kolla and Mishra, 2018). In healthy individuals, for example, circulating levels of AEA have been found to be inversely associated with anxiety (Dlugos et al., 2012) and need for positive reward feedback (Redlich et al., 2021). Here, we observed significant differences in levels of DEA in both male and female mice between Dom and Sub groups. Although DEA was identified about 30 years ago (Hanus et al., 1993), it has yet to be adequately studied. DEA showed affinity to the CB1 receptor at concentrations close to structurally related compound AEA (IC_50_ 190 nM vs. 160 nM, respectively) (Felder et al., 1993) and produced similar responses in a set of behavioral assays for cannabinoid-like effects (Barg et al., 1995). DEA has also been demonstrated to be an agonist of the vanilloid receptor (TRPV1) (Movahed et al., 2005) and, along with related endocannabinoids, is produced in the CNS by neurons, microglial cells, and astrocytes (Walter et al., 2002). The difference between DEA and AEA is in the fatty acid component of the molecule. Endocannabinoids mostly consist of fatty acids coupled to ethanolamide, glycerol, and amino acids. In the main 2 endocannabinoids AEA and 2-AG, the fatty acid is arachidonic acid (AA), while in DEA the fatty acid is docosatetraenoic acid (DA), both of them are polyunsaturated fatty acids (PUFA).

PUFA family (Spector, 1999) is typically classified based on the number and position of their double carbon bonds (carbons number:double bonds number). In addition to AA (20:4), the parent fatty acid of AEA and 2-AG, this family includes other fatty acids such as ɑ-linolenic acid (ALA; 18:3), EPA (20:5), DHA (22:6), linoleic acid (LA; 18:2), DA (22:4), and others. Dysregulation of PUFA homeostasis was found to worsen the course of mental illness and is associated with neurodegenerative processes (Petermann et al., 2022; Stachowicz, 2023). In a recent review of meta-analyses, PUFA supplementation was found to be partially favorable in treatment of anxiety, depression, attention-deficit/hyperactivity disorder (ADHD), autism spectrum disorder (ASD), dementia, mild cognitive impairment, Huntington’s disease, and schizophrenia (Gao et al., 2022). Therefore, we may infer that distinct behavioral characteristics of Dom and Sub mice may partially stem from different brain levels of specific PUFA measured here. The concentrations of PUFA in the body are typically mirrored by the levels of their ethanolamide derivatives. For example, supplementing milk formulas with AA and DHA raised the levels of the corresponding NAEs, AEA, and DHEA, in certain brain regions in piglets (Banni and di Marzo, 2010). In addition, feeding with an AA-rich diet produced increased AEA whole-brain concentrations in mice (Banni and di Marzo, 2010). The differences in the levels of the parental fatty acids (DA and EPA) in brains of Dom and Sub mice hint that imbalance in the regulation of endocannabinoid biosynthesis in these behaviorally-distinct populations may occur upstream of the endpoint ethanolamide derivatives. Our assumption was further confirmed by differential mRNA expression of genes encoding the enzymes (FADS2 and ELOVL5) responsible for PUFA biosynthesis in the brains of Dom and Sub mice. An increased expression of enzymes involved in the desaturation and elongation of PUFA in Sub mice aligns with the results achieved by lipidomic profiling and further provides an explanation for the observed rise in DEA and related metabolites in these mice. It is noteworthy that *Fads2* transcription, which is involved in fatty acid desaturation and arachidonic acid synthesis, was upregulated in high-fat diet treated mice exhibiting depressive-like behavior (Yu et al., 2021). ELOngation of Very Long-chain fatty acids (ELOVLs) are enzymes involved in the initial condensation reaction necessary to elongate fatty acids (Sassa and Kihara, 2014). ELOVL5 can condense a wide range of PUFA, including condensation of linoleic acid to produce arachidonic acid, α-linolenic acid to produce eicosapentaenoic acid, and stearidonic acid to produce docosahexaenoic acid (Shikama et al., 2015). Furthermore, ELOVL5 is highly expressed in the central nervous system and mutations in the gene were found to be the cause of spinocerebellar ataxia type 38 (SCA38), a rare autosomal neurological disease characterized by gait abnormality, dysarthria, dysphagia, hyposmia, and peripheral neuropathy (Balbo et al., 2021). While reduction in ELOVL5 expression has been linked to worsened prognosis in breast estrogen receptor-positive cancer patients (Kieu et al., 2022), its role in behavior regulation is still unclear. The observed increased expression of ELOVL5 and FADS2 in Sub mice, together with the elevation of the parental DA and EPA levels, as well as with DEA and related metabolites, suggest that the signaling pathways mediated by these molecules are of importance for social behavior and personality manifestations.

DEA was previously shown to produce classical cannabinoid-like effects at higher concentrations (15–60 mg/kg) (Barg et al., 1995). Indeed, we found that, among tested doses (5, 10, 15 mg/kg), significant antinociceptive effects were observed at a dose of 15 mg/kg, however only in Sub mice. While no anxiolytic-like effects were observed, 15 mg/kg induced locomotory activity in EPM assessments. Interesting, that this phenomenon was also examined in Sub, but not in Dom mice. However, dose as low as 5 mg/kg had no effect in either hot plate or EPM in Sub and Dom mice. Our previous observations demonstrated that Dom and Sub mice differentially respond to psychotropic agents (Nesher et al., 2013; Murlanova et al., 2021). Acute administration of the selective serotonin reuptake inhibitor paroxetine produced pronounced antidepressant-like effects in Sub, while paradoxical (frenetic) activity was observed in Dom (Nesher et al., 2013). Differential responses were also observed with mood stabilizers and addictive compounds (Nesher et al., 2013; Murlanova et al., 2021). We assume, that differential responses of Dom and Sub mice to DEA, observed in this study, may occur due to distinct brain-regions specific patterns of monoamines of these mice (Murlanova et al., 2021). In previous studies, an interaction was found between nociception and negative social interaction (defined as “social pain” (Sturgeon et al., 2020), moreover, they are processed by a shared neural circuitry (Zhang et al., 2019). Many therapeutics, such as cannabinoids, opioids, antidepressants, etc. also share the effect on both, affecting mutually pain perception and mood/behavior (Negri et al., 1996; Greenwald and Stitzer, 2000; Duman et al., 2004; Bomholt et al., 2005; Hache et al., 2011). Indeed, our results show that the treatment with DEA may exert modularity effect on social (hierarchy-based) personality. Research has shown that social hierarchy is linked to personality traits in both animals and humans (Colléter and Brown, 2011; David et al., 2011; Dasgupta et al., 2022; Vékony et al., 2022). For example, dominant individuals tend to be more assertive, confident, and proactive (Blanchard et al., 1988; Salonen et al., 2022), while submissive individuals are often more anxious, passive, and reactive (Catarino et al., 2014; Zaffar and Arshad, 2020). In addition, social hierarchy can shape individual behavior and physiology (Schmid Mast, 2010; Flota-Bañuelos et al., 2019) including stress responses (Knight and Mehta, 2017; Karamihalev et al., 2020), brain activity (Breton et al., 2014; Williamson et al., 2019a), and gene expression (Pohorecky et al., 2004; Horii et al., 2017; Lea et al., 2018; Williamson et al., 2019b).

As DEA is higher in the brain of Sub mice, it would be straightforward to assume that its administration may enhance submissiveness, but this assumption was not confirmed by pharmacological studies, where DEA administration was not able to reduce submissiveness of Sub animals. We may suggest that elevation of DEA in the brain of Sub mice acts as a compensatory mechanism to counterbalance submissiveness-associated features including enhanced stress vulnerability, anxiety, and social deficits (Gross and Pinhasov, 2016; Yanovich et al., 2018). Moreover, in support of this hypothesis, we clearly demonstrate here that DEA further induced dominance levels of selectively-bred dominant mice. This phenomenon should be further evaluated as it suggests that the ECS is important in regulation of social behavior. It is possible that DEA may invoke activation of a signal transduction pathway, distinct from canonical cannabinoid receptor-mediated neurotransmission. This pathway would be activated by DEA at levels that do not produce the classical CB1-mediated psychotropic and antinociceptive effects. This hypothesis is partially supported by gene transcription analysis of Sub and Dom mouse brain, which did not show any significant differences in the expression levels of cannabinoid system related genes *Cnr1*, *Cnr2*, *Trpv1*, *Gpr55*, and *Gpr18*.

The diverse endocannabinoid profiles observed in behaviorally-distinct populations of mice reported here, in addition to the phenotype-dependent effect of DEA on nociception, locomotion and modulation of dominance, highlight the role of the ECS in regulating social behavior. By unraveling the intricate relationship between endocannabinoids and social behavior, this research contributes to a deeper understanding of the neurobiological foundations of social behavior and holds promise for advancing our knowledge of psychiatric disorders characterized by social dysfunctions.

## In memoriam

This article is dedicated to the memory of Professor Raphael Mechoulam (1930−2023), a pioneer of cannabinoid research, who was actively participating in this project until his last days.

## Figures and Tables

**Table:** 

**2-AG**	2-Arachidonoyl Glycerol
**2-LG**	2-Linoleoyl Glycerol
**2-PG**	2-Palmitoyl Glycerol
**AEA**	Arachidonoyl ethanolamide (anandamide)
**ARA-G**	Arachidonoyl Glycine
**ARA-S**	Arachidonoyl Serine
**DA**	Docosatetraenoic acid
**DEA**	Docosatetraenoyl Ethanolamide
**DEEA**	Docosaenoyl Ethanolamide
**DHEA**	Docosahexaenoyl Ethanolamide
**DSEA**	Docosaenoyl Ethanolamide
**DSR**	Dominant-Submissive response
**DTEA**	Docosatrienoyl Ethanolamide
**ECS**	Endocannabinoid system
**EPA**	Eicosapentaenoic acid
**EPEA**	Eicosapentaenoyl Ethanolamide
**EPM**	Elevated Plus-Maze
**ETEA -5(Z), 8(Z), 11(Z)**	Eicosatrienoyl Ethanolamide
**LauEA**	Lauroyl Ethanolamide
**LEA**	Linoleoyl Ethanolamide
**MEA**	Myristoyl Ethanolamide
**NAE**	N-acyl ethanolamide
**OA**	Oleoyl Alanine
**OEA**	Oleoyl Ethanolamide
**OG**	Oleoyl Glycine
**OS**	Oleoyl Serine
**OT**	Oleoyl Taurine
**PDEA**	Pentadecanoyl Ethanolamide
**PEA**	Palmitoyl Ethanolamide
**PUFA**	Polyunsaturated Fatty acid
**SEA**	Stearoyl Ethanolamide
**THC**	Tetrahydrocannabinol
**VEA**	Vaccenoyl Ethanolamide

## Data Availability

The original contributions presented in the study are included in the article/Supplementary Material, further inquiries can be directed to the corresponding author.
