# Supplementary material for: Endocannabinoid basis of personality—Insights from animal model of social behavior
Source: Front Pharmacol. 2023 Aug 16;14:1234332. doi: 10.3389/fphar.2023.1234332 (PMC10468576; doi:10.3389/fphar.2023.1234332)
Supplement: Supplementary file 1 [file Presentation1.PPTX]

## Slide 1
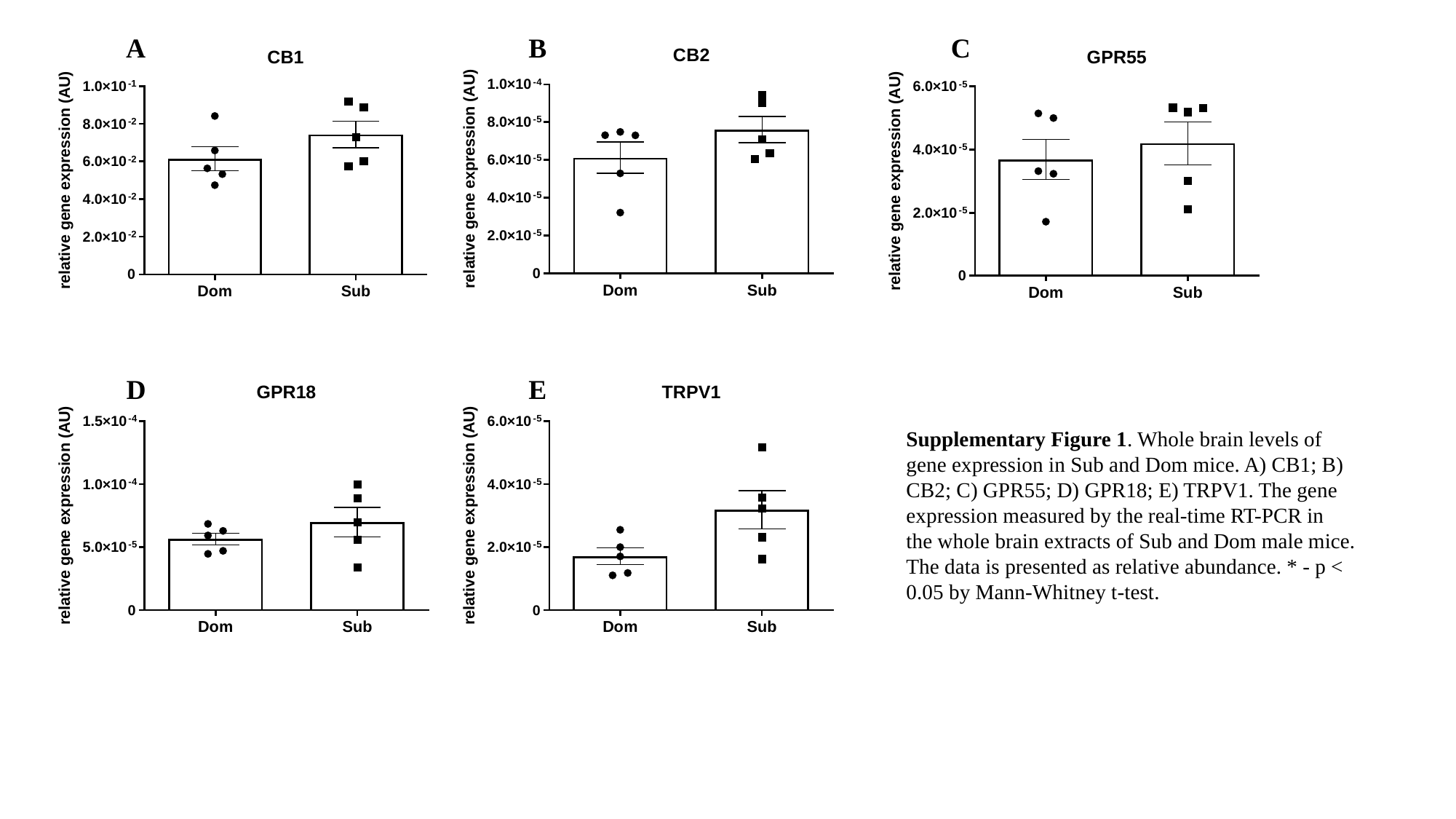

A
B
C
D
E
Supplementary Figure 1. Whole brain levels of gene expression in Sub and Dom mice. A) CB1; B) CB2; C) GPR55; D) GPR18; E) TRPV1. The gene expression measured by the real-time RT-PCR in the whole brain extracts of Sub and Dom male mice. The data is presented as relative abundance. * - p < 0.05 by Mann-Whitney t-test.

## Slide 2
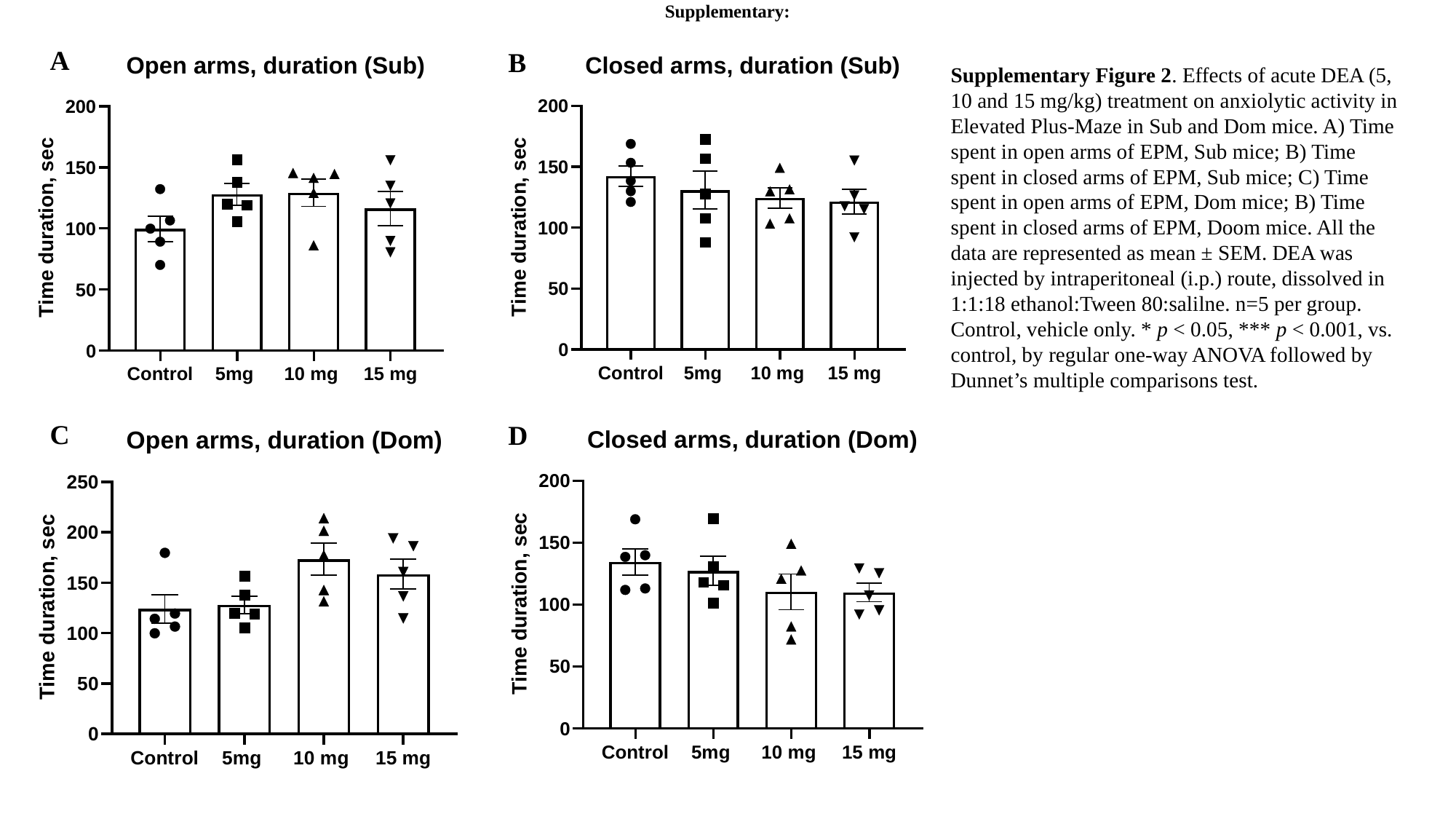

Supplementary:
Supplementary Figure 2. Effects of acute DEA (5, 10 and 15 mg/kg) treatment on anxiolytic activity in Elevated Plus-Maze in Sub and Dom mice. A) Time spent in open arms of EPM, Sub mice; B) Time spent in closed arms of EPM, Sub mice; C) Time spent in open arms of EPM, Dom mice; B) Time spent in closed arms of EPM, Doom mice. All the data are represented as mean ± SEM. DEA was injected by intraperitoneal (i.p.) route, dissolved in 1:1:18 ethanol:Tween 80:salilne. n=5 per group. Control, vehicle only. * p < 0.05, *** p < 0.001, vs. control, by regular one-way ANOVA followed by Dunnet’s multiple comparisons test.
A
B
C
D
